# 2492: Leveraging CTSA informatics capacity to expand global health engagement and research capacity in Latin America and the Pacific

**DOI:** 10.1017/cts.2017.80

**Published:** 2018-05-10

**Authors:** Timothy De Ver Dye, Thomas Fogg, Margaret Demment, José Pérez-Ramos, Scott McIntosh, Deborah Ossip, Angela Sy, Carmen Velez Vega, Karen Peters, Haq Nawaz

**Affiliations:** University of Rochester Medical Center, Rochester, NY, USA

## Abstract

OBJECTIVES/SPECIFIC AIMS: The objective of this partnership was to create a global network of clinical and public health researchers and communities conducting technology-assisted research in noncommunicable disease. METHODS/STUDY POPULATION: The University of Rochester’s Clinical and Translational Science Institute (CTSI) has successfully leveraged the informatics core’s capacity into an emerging network of organizations that focus on technology and health in settings outside of the mainland United States. The CTSI coordinated with another NIH-funded infrastructure program [the RCMI Translational Research Network (RTRN)] to identify partner institutions interested in technology and health. RTRN identified the University of Puerto Rico and the University of Hawaii, both of which serve as hubs for common research interests in technology and health throughout the Caribbean and the Pacific. This network was formalized as the CDC’s Coordinating Center for its Global and Territorial Health Research Network (the “Global Network”), with additional US partners (Yale, University of Illinois at Chicago, University of North Caroline Chapel Hill, and the University of South Florida) within a wider scope of the CDC’s Prevention Research Centers (PRC) program. RESULTS/ANTICIPATED RESULTS: Through combining 2 main NIH-funded research infrastructure networks (CTSA and RTRN), with a large CDC-funded PRC, the University of Rochester’s Informatics Core was successful in establishing a new productive global health network throughout Latin America and the Caribbean, and in the Pacific, garnering additional research support from NIH Fogarty and other programs. The resulting network not only supports locally-important research in technology and health on compelling health issues (eg, diabetes, ZIka, participation in research), but also facilitates community engagement through local partnerships and the cores of the involved networks. In addition, much of the information and communications technology (ICT)-related research and learnings from the Global Network activity is immediately applicable to populations in the United States, served by the various collaborative networks. In total, while new, the Global Network supports a wide range of projects and engagements throughout the world that expand local informatics capacity and use of technology in the research process and to address global health problems, further enhancing the CTSI’s informatics core to serve the needs of its own constituency and promote research engagement with technology within this population. Local research collaborative projects reinforce the utility of the network and its resources, evidenced by tools, publications, partnerships, and conference presentations that have arisen. Lessons to date from this Global Network collaboration include: specific global research projects provide opportunities for partnership building and meaningful collaboration, team science is of central importance in distributing the work of the network, synergy is multidirectional with expertise and need flowing in all directions, and project team members in all locales learned and contributed substantially in ways that carried into their other responsibilities. DISCUSSION/SIGNIFICANCE OF IMPACT: The overall partnership has created opportunity for South-South collaboration, for adaptation of projects among locales, and has helped boost reputational value for all partners involved. Implications for other CTSA awardees include: global collaboration can serve core research and technical needs for the CTSA itself and its local partners, CTSA status can be leveraged to access resources to support local research, and collaboration in other federally-funded research networks helps expand the insight, scope, and potential for new research.

